# Semi-Supervised Learning for Defect Segmentation with Autoencoder Auxiliary Module

**DOI:** 10.3390/s22082915

**Published:** 2022-04-11

**Authors:** Bee-ing Sae-ang, Wuttipong Kumwilaisak, Pakorn Kaewtrakulpong

**Affiliations:** 1 Electrical and Engineering, King Mongkuts University of Technology Thonburi, Thung Khru, Bangkok 10140, Thailand; 2 Tesla Inc., Austin, TX 78725, USA; pkktao@gmail.com

**Keywords:** defect segmentation, deep learning, semi-supervised learning

## Abstract

In general, one may have access to a handful of labeled normal and defect datasets. Most unlabeled datasets contain normal samples because the defect samples occurred rarely. Thus, the majority of approaches for anomaly detection are formed as unsupervised problems. Most of the previous methods have typically chosen an autoencoder to extract the common characteristics of the unlabeled dataset, assumed as normal characteristics, and determine the unsuccessfully reconstructed area as the defect area in an image. However, we could waste the ground truth data if we leave them unused. In addition, a suitable choice of threshold value is needed for anomaly segmentation. In our study, we propose a semi-supervised setting to make use of both unlabeled and labeled samples and the network is trained to segment out defect regions automatically. We first train an autoencoder network to reconstruct defect-free images from an unlabeled dataset, mostly containing normal samples. Then, a difference map between the input and the reconstructed image is calculated and feeds along with the corresponding input image into the subsequent segmentation module. We share the ground truth for both kinds of input and train the network with binary cross-entropy loss. Additional difference images can also increase stability during training. Finally, we show extensive experimental results to prove that, with help from a handful of ground-truth segmentation maps, the result is improved overall by 3.83%.

## 1. Introduction

Defect detection/segmentation has wide applications in the quality control process in manufacturing, medical diagnosis, and quality inspections. It aims to produce, given an input image, a segmentation map that corresponds to the defect area. With the increasingly stringent requirements for the quality of products, this process has become an indispensable part of many manufacturing methods. However, manual inspection by humans is slow, expensive, and error-prone, resulting in more and more automated inspection systems running in the production lines. Most of the existing research formulates this problem as anomaly detection with the assumption that normal examples are the majority in the total collection of data. The distribution of normal samples is often learned in an unsupervised manner and defect detection is achieved by discovering out-of-distribution samples [1,2,3,4]. In some applications, especially high-precision production, the location of defects can be critical to the product. Some areas on a product’s surface are strictly not allowed to have any small defects on them. It is more accurate to capture this kind of defect by machine. Furthermore, the detection information can be an indicator of potential machine operation failure. Noticing problems early aids in maintenance cost reduction.

With the advent of deep learning, attention has been paid to exploiting the strong feature learning ability of neural networks for many applications [5,6], including anomaly detection [2,4].

In most cases, the number of defect samples is extremely minimal, although the number of good samples is plentiful. An anomaly detector is often designed under one-class classification using normal data only. To formulate the anomaly detection as a classification problem, one-class SVMs [7] have been proposed to learn deep feature mapping such that normal samples are embedded within a hypersphere and anomalous samples are determined by a threshold.

A deep one-class method [4] employs a similar concept, which uses a hypersphere to encapsulate the normal features. These features are extracted by convolutional layers to obtain spatial information. However, a hypersphere is not always the best choice for modeling complex manifolds, which are almost always inescapable.

Another line of works employs an autoencoder (AE) [8,9] to capture the main variance of normal samples in a similar way to PCA, while benefiting from the power of non-linear and deep feature learning capabilities.

Typically, we train models to capture patterns from normal instances and establish anomalies if the test example cannot be properly represented by these models. Anomalies are identified by comparing the input and reconstructed samples. Other works model the manifold of normal samples by training a generative adversarial network (GAN) [10] and anomalous samples are identified via measuring the deviation from the normal manifold [2]. Among these alternatives, autoencoder-based approaches have demonstrated superiority due to the clear assumption of identifying the normal patterns by dimension reduction, low computation cost, and the superior performance for anomaly detection benchmark [3]. More specifically, an autoencoder extracts the typical patterns of high-dimensional data through the bottleneck latent layer. The compact coding has another benefit in preventing the network from simply copying an input. Then, the high reconstruction error is interpreted as an anomaly score. For anomaly segmentation, the per-pixel error is computed to indicate defect pixels. However, the methods described above mainly rely on reconstruction. With the defect area filtered out, obtaining a perfect reconstructed output is very difficult.

Despite the success of existing AE-based anomaly detection methods, there are still a few challenges that plague the unsupervised methods. Firstly, an AE requires careful tuning of the compression rate, e.g., the dimension of the bottleneck layer. An overly low compression rate may limit the ability to capture the inherent variation within normal samples and the opposite would capture too much variation with a risk of reconstructing the anomalies that totally fails the anomaly detection. Secondly, for anomaly segmentation, AE-based methods rely on the difference between input and reconstruction; therefore, the segmentation map may not be accurate at the boundaries and is prone to misalignment and variation of visual patterns. As a result, existing AE-based methods often require careful parameter tuning and generally achieve low precision.

Contrary to the common assumption that anomaly detection models are trained in a totally unsupervised fashion, it is often possible to obtain a few labeled samples, e.g., in IC manufacturing some anomalous chips are identified and can be manually labeled. Therefore, this raises the question of whether anomaly detection can be further improved by a limited number of labeled samples, which method is termed semi-supervised anomaly detection in this work. To the best of our knowledge, there are very few works addressing such a scenario. Among these, L. Ruff et al. [11] extended the deep one-class classification [4] by incorporating additional losses defined upon labeled data, but the method could not be trivially adapted to anomaly segmentation tasks. Subsequent work proposed a weakly supervised approach for anomaly segmentation [12]; however, it failed to directly utilize the labeled anomalous segmentation map.

To tackle the aforementioned challenges, we propose a semi-supervised anomaly segmentation approach.

Although there is a lot of diversity in the distribution of anomaly patterns, providing a few defect samples to the model can help it acquire more discriminative features since they come from real samples. Our approach consists of two parts. The first part, an autoencoder, is trained to reconstruct images upon all available data in a similar way to [13]. For the limited labeled anomalous samples, both original and reconstructed images are fed into the segmentation network to produce a final segmentation map.

**Figure 1:**
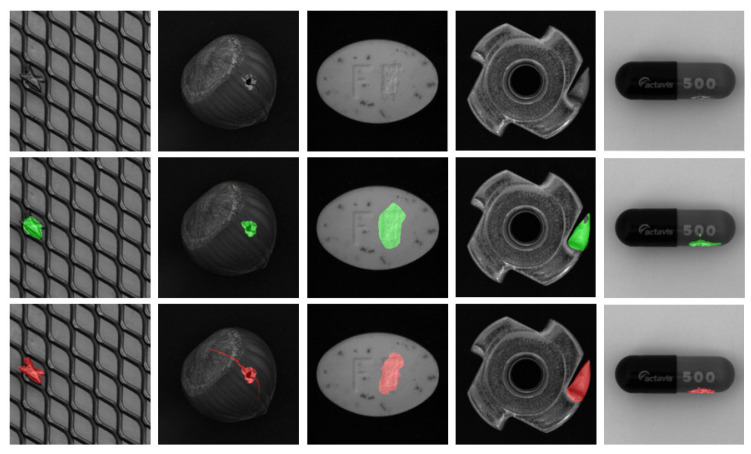
Examples of the objects and their segmentation maps. (**Top row**) Original input. (**Middle row**) Our method’s results. (**Last row**) Segmentation ground truth.

In this way, the model can further fine-tune results from the autoencoder. To avoid overfitting, we apply strong data augmentation to expand the variation of training images. Some selected results are depicted in Figure 1.

To summarize, we make the following contributions in this work:

- To the best of our knowledge, this is the first work addressing defect segmentation in a semi-supervised manner. It requires only 2% labeled training samples whereas it achieves a 20% improvement over existing unsupervised approaches on the publicly available MVTec dataset [3].
- We propose a novel framework by first exploiting an autoencoder to learn indiscriminately from all unlabeled data. Then, a Unet segmentation network is trained on labeled data with both classification and consistency losses. From our experimental results, this method outperforms all state-of-the-art generic semi-supervised methods.

The rest of the paper is organized as follows. In Section 2, we give a brief overview of related works on semantic segmentation with a focus on semi-supervised learning. Section 3 then describes our model and experimental results are given in Section 4. Finally, the conclusions are summarized in Section 5.

## 2. Related Work

### 2.1. Anomaly Detection

Anomaly detection aims to discover unusual patterns from normal ones and is often formulated in an unsupervised fashion. Traditional methods include PCA, cluster analysis [14], and one-class classification [1]. With the advent of deep learning, stronger feature learning capability has been employed to avoid complicated feature engineering and kernel construction. This leads to novel anomaly detection methods based on generative adversarial networks (GANs) [2,15] and autoencoders [13]. Among the former line of works, the AnoGAN [2] was proposed to learn the manifold of normal samples and anomalous samples could not be perfectly projected onto the normal manifold by the generator learned from normal samples only. However, this method requires expensive optimization for detecting abnormal samples and training GAN is prone to some well-known challenges including instability, mode collapse, etc. Among the autoencoder-based approaches, P. Bergmann et al. [13] adopted an SSIM metric as the similarity measure between input and reconstructed images. It was demonstrated to be better than the traditional L2 norm for detecting anomalous pixels for anomaly segmentation, which is the major concern in this paper.

Nevertheless, the SSIM is well suited to clearly structured object images such as fabric. For object-centric photos, it does not produce satisfactory results.

### 2.2. Semantic Segmentation

Typically, a classification network reduces the size of the output when it goes deeper. This affects the performance of segmentation tasks. The fully convolutional network, created by J. Long et al. [16], is one of the pioneer works in semantic segmentation tasks. Their previous layer features are summed with the output of the layer before upsampling and finally, prediction. Later, O. Ronneberger et al. [17] devised the Unet architecture designed to concatenate more feature from previous layers, introducing richer raw features from early layers. L. Chen et al. [18] employed an atrous convolution layer in DeepLab to extract context in a wider receptive field while preserving the same computation cost. Y. Liu et al. [19] performed knowledge distillation, training small networks by making use of large networks for each pixel separately. Context is known to be crucial for semantic segmentation. H. Ding et al. [20] and Y. Zhou et al. [21] used context information to further enhance the network performance. However, most existing works focus on detecting semantic segmentation from object-centric natural images with fully supervised labeled samples. This does not generalize well in anomaly localization with limited defect labeled samples.

### 2.3. Semi-Supervised Learning

A deep neural network trained with full supervision needs a huge amount of data. It is known to be sensitive to labeled training data with supervised tasks, e.g., classification, regression, segmentation, etc. However, in some cases, labeled data are costly and time consuming to collect. J. E. Van Engelen and H. H. Hoos [22] give good overall details of semisupervised methods. Since unlabeled data are much easier to gather, semi-supervised approaches are designed to extract knowledge from unlabeled data along with a handful of labeled data.

To learn generalizable models with limited labeled data, semi-supervised learning [23,24] has been investigated to exploit additional large amounts of unlabeled data, with an extensive review given by [22]. Although there has been substantial progress in image recognition [24], and semantic segmentation [25], etc., have been witnessed, SSL is rarely studied in the context of anomaly detection. It is often reasonable to assume that a small number of anomalous samples are identified and labeled with ground-truth and SSL is the most appropriate approach towards this setting. As the first attempt to formulate anomaly detection in a semi-supervised manner, deep semi-supervised anomaly detection [11] added supervision loss for labeled data into a one-class network [4]. However, there is no trivial way to generalize such an approach to anomaly segmentation tasks, where identifying anomalies for individual pixels is more difficult than classifying a whole image. In this work, we proposed to combine an autoencoder and a segmentation network to simultaneously exploit very few labeled and a large amount of unlabeled images for anomaly segmentation.

## 3. Methodology

Given *N* (mostly *N* $I_{1},\ldots ,I_{N}$ and *M* labeled samples $\left({\tilde{I}}_{1},{\tilde{Y}}_{1}\right),\ldots ,\left({\tilde{I}}_{M},{\tilde{Y}}_{M}\right)$), where ${\tilde{y}}_{ij}$ could be 0 or 1, denoting normal and defective pixels (i,j) in each image, the task is to learn a model that detects anomalous regions in an image.

**Figure 2:**
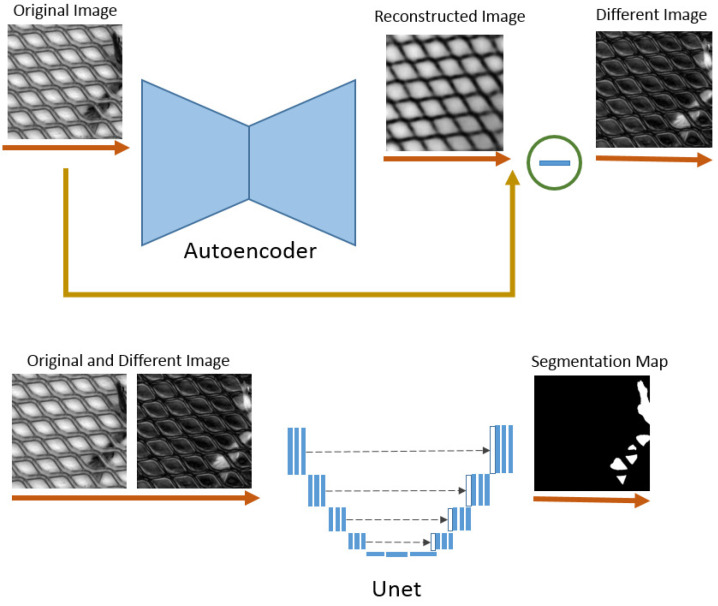
The overall architecture: Input unlabeled data are fed into a pre-trained autoencoder. The difference image from reconstructed and original images is then calculated. For training Unet to predict the segmentation map, both input and difference images are concatenated to obtain better results.

Our auto-encoder takes an image *I* as input with size W×H×C, where *C* is the number of channels, learns latent embedding and then reconstructs the image. A classifier (Unet) is adopted, and is then applied to produce a segmentation map W×H×K, where *K* is the number of classes. For our task, K is equal to one due to the nature of binary segmentation, defect, and non-defect classes. The overall framework is summarized in Figure 2.

### 3.1. Unsupervised Feature Learning

Autoencoders perform dimensionality reduction by encoding the input into a far lower dimension, which is called the latent representation. Fascinatingly, during the process of dimensionality reduction, outliers are identified. This latent representation will have far less dimension that would store only the main pattern. The main pattern is considered by the frequency of appearance of that pattern. Therefore, when the network tries to reconstruct the anomaly, which occurs rarely, it fails. Principal component analysis (PCA) is known as the classical useful tool to detect outliers by dimensionality reduction. However, PCA transforms the input into latent representation and transforms it back linearly. In contrast, the autoencoder techniques can perform non-linear transformations with their non-linear activation function following each convolutional layer. It is more efficient to train several layers with an autoencoder, rather than training one huge transformation with PCA. The autoencoder techniques are thus more suitable when the data problems are complex and non-linear in nature.

**Figure 3:**
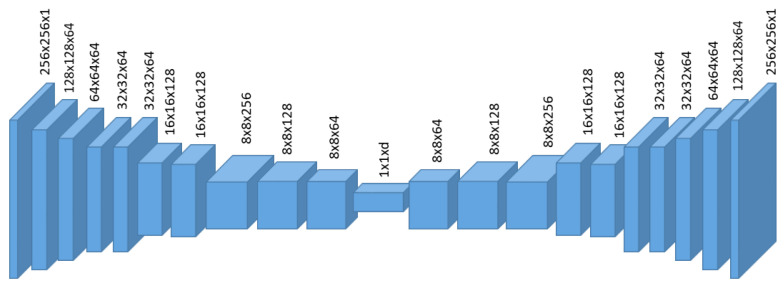
Details of encoder in our autoencoder architecture.

Given a large amount of unlabeled data, we employ an autoencoder (AE) as shown in Figure 3 to learn normal feature patterns in an unsupervised fashion. Given a proper bottleneck dimension, it has been demonstrated that an AE will only capture the major variations of normal data, hence the reconstructed images would remove the outlier patterns [13]. One is able to directly compare the difference between input and reconstruction for anomaly segmentation. Specifically, an AE consists of an encoder function $E:R^{W\times H\times C}\overset{}{\to }R^{d}$ and a decoder function $D:R^{d}\overset{}{\to }R^{W\times H\times C}$, where *d* denotes the dimension of the latent vector, which is viewed as a compact representation of an input image. Allowing d≪W×H×C encourages the architecture to avoid trivial identity mapping from input to output and prune out noisy signals. In general, the process can be expressed as,

(1) $$\hat{I}=D\left(E\left(I\right)\right)=D\left(z\right)$$

(2) $$MSE=\frac{1}{NWH}\sum_{k=1}^{N}\sum_{i,j}^{W,H}{∥I_{ij}-{\hat{I}}_{ij}∥}_{2}^{2}$$

**Figure 4:**
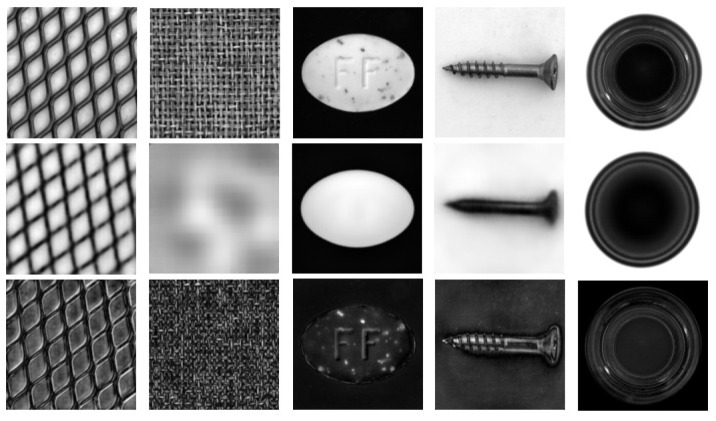
(**Top**) Original image. (**Middle row**) Reconstructed images from AE. (**Last row**) Difference images. The magnitudes of misalignment pixels are obviously high and result in false detection.

In order to reconstruct the input image in a pixelwise manner, the encoder will encode the major variations in input data and, as a result, the reconstructed image will be largely defect-free. If the input image contains any anomaly, it will be ideally detected by measuring the absolute difference between input and reconstruction as,

(3) $$D_{ij}=\left|I_{ij}-{\hat{I}}_{ij}\right|$$

where *D* denotes the difference image. Some of results are presented in Figure 4. However, the accuracy of the difference image heavily depends on the intra-class (normal to normal) vs. inter-class (normal to abnormal) variation and a proper selection of the bottleneck layer dimension. The former depends on the data distribution and the latter is not trivial to select. To resolve these issues, we exploit the limited labeled samples to train a discriminative model for more accurate prediction.

### 3.2. Semi-Supervised Anomaly Segmentation

Given extremely limited labeled anomalous samples, we specify a Unet as the segmentation model. Because of possessing huge parameters, the Unet can easily overfit this limited dataset. To avoid overfitting, the model requires additional knowledge for the segmentation task. First, we utilize an autoencoder to learn the pattern from good samples. In this way, the model can detect the anomaly area. This does, however, rely greatly on the quality of the reconstruction. We solve this limitation by introducing the Unet to further localize the defect regions using the result calculated from the autoencoder output. Unet is one of the popular architecture choices for semantic segmentation tasks. The network comprises a contracting path and an expansive path, also perceived as encoder and decoder. There are skip links between these two parts to propagate the context information to successive layers. This gives the architecture a u-shaped form. It benefits from learning feature maps at multiple scales to capture both the detailed structural information and rough spatial extent. Because our work occasionally necessitates the detection of small defect regions, the features transmitted from the very earliest layers may provide useful information for capturing these types of defects. A standard Unet [17] is adopted here.

The contracting path consists of the repeated application of two 3 × 3 convolution layers with padding to preserve the size of the output, each followed by a rectified linear unit (ReLU). The 2 × 2 max pooling operation with stride 2 is employed for downsampling the layer after every two convolutional layers. Then, the number of feature channels is doubled after each downsampling step. For an expansive path, it consists of an upsampling of the feature map followed by a 2 × 2 convolution (“up-convolution”). The number of feature channels is reduced to half in this step. The features from the contracting path are propagated to the expansive path by concatenating with the corresponding feature map. Then, each is followed by two 3 × 3 convolutions and a ReLU. At the final layer, a 1 × 1 convolution layer maps 64 feature channels to the desired number of classes for each pixel, which is two classes in our case.

Importantly, we concatenate the raw input image and the different image obtained from the auto-encoder. In this work, Unet maps an original image and the corresponding difference image, the result from the pre-trained autoencoder, to the segmentation ground truth. The advantages of the difference image is that it contains the guidance of possible defect regions. This is reasonable because the defect areas have been potentially captured by the difference image and the Unet will benefit from feeding with features with richer prior information. Let ϕ((·)W,b) be the function represented by our Unet network; we have

(4) $$\hat{Y}=\phi \left(\left(\left[I;D\right]\right)W,b\right)$$

where ; denotes a channel-wise concatenation and W,b indicates the parameters, weights, and biases of network. The final input is channel-wise concatenation of the original raw image *I* and difference image *D*. Finally, we define a pixel-wise sigmoid cross-entropy loss as follows,

(5) $$L_{CE}=\sum_{k}^{M}\sum_{i,j}^{W,H}\left[{\tilde{Y}}_{ijk}\log{\hat{Y}}_{ijk}+\left(1-{\tilde{Y}}_{ijk}\right)\log\left(1-{\hat{Y}}_{ijk}\right)\right]$$

where *M* is the number of labeled samples.

### 3.3. Data Augmentation

To avoid overfitting from limited labeled data, data augmentation is performed during the training stage. We first normalize all pixel values into the range [0, 1]. The input images are downsampled to 256×256 pixels. We apply random rigid affine transformations, rotation and translation, to increase the variation of the training set. Random flipping along *X* and *Y* axes is also applied wherever possible. In the affine case, a pixel coordinate is transformed by

(6) $$\left[\begin{array}{c} {\hat{x}}_{i}\\ {\hat{y}}_{i} \end{array}\right]=A_{\theta }\left(\begin{array}{c} x_{i}\\ y_{i}\\ 1 \end{array}\right)=\left[\begin{array}{ccc} \theta_{11} & \theta_{12} & \theta_{13}\\ \theta_{21} & \theta_{22} & \theta_{23} \end{array}\right]\left[\begin{array}{c} x_{i}\\ y_{i}\\ 1 \end{array}\right]$$

where $\left({\hat{x}}_{i},{\hat{y}}_{i}\right)$ and $\left(x_{i},y_{i}\right)$ represent input and transformed coordinates, respectively. $A_{\theta }$ is an affine transformation matrix for rigid body transformation and can be written as

(7) $$A_{\theta }=\left[\begin{array}{ccc} cos\alpha & -sin\alpha & t_{x}\\ sin\alpha & cos\alpha & t_{y} \end{array}\right]$$

where α, $t_{x}$ and $t_{y}$ control rotation angle and translation distance, respectively. This augmentation is randomly and differently performed in every training batch.

**Figure 5:**
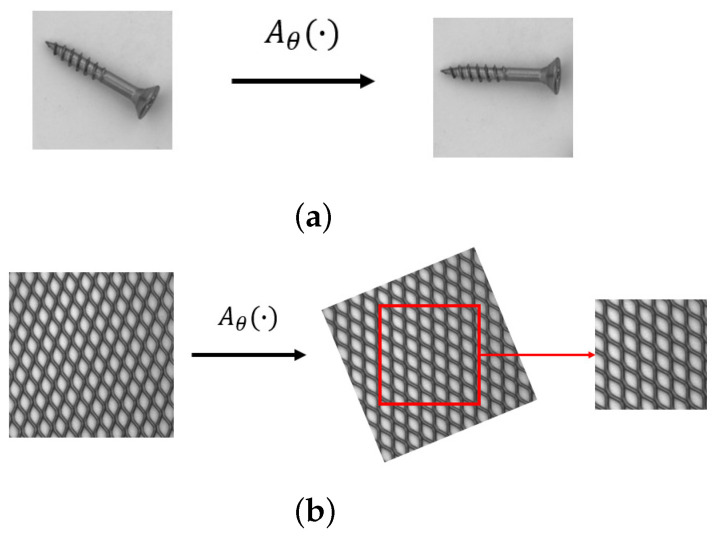
(**a**) Augmentation for non-texture object categories and (**b**) augmentation for texture object categories.

For texture object categories, we down-size the image into 512×512 before applying random transformation. Finally, we crop the transformed image into a size of 256×256 pixels for training. The effect of the mentioned augmentation in both non-texture and texture categories is displayed in Figure 5.

## 4. Experiment

### 4.1. Dataset

MVTec Dataset [3]: This dataset contains 15 object categories, including 10 non-texture object categories and 5 texture object categories. The texture categories consist of regular (carpet, grid) or random (leather, tile, wood) textures, while the non-texture categories represent various types, such as rigid with a fixed appearance (bottle, metal nut), deformable (cable), and natural variations (hazelnut).

Each category contains around 60–300 normal samples for training and 30–400 normal and defect samples for testing. We augment the training set by selecting a couple of defect samples from the testing set. For the supervised labeled sample, we pick an image from each defect type. Therefore, the percentage of labeled samples ranges from 2 to 4% in each of the categories. Table 1 summarizes the details of the number of samples in all categories.

### 4.2. Implementation Details

Both the autoencoder and Unet were trained separately using the Adam optimizer. The learning rate was initially set to 0.0001 with a decay rate equal to 0.8 in every 30 epochs, for a total of 200 epochs. The batch size was set to 16 while the latent vector dimension was set to 500 for the autoencoder. The code was implemented in TensorFlow and experiments were run on a server equipped with an NVIDIA 1080Ti GPU (12 GBs). All methods were evaluated with the same test dataset as shown in Table 1.

The autoencoder was trained prior to training the Unet. We utilized the autoencoder to obtain a good sample pattern from an unlabeled dataset. The Unet was then used to further localize the defect regions using data calculated from the autoencoder output combined with the labeled dataset. Both were trained with augmentation as explained in Section 3.3.

### 4.3. Evaluated Methods

We compared our results against those from both unsupervised anomaly segmentation methods and generic semi-supervised methods. For comparisons to unsupervised methods, we first compared with the traditional dimension reduction method, PCA. We then reproduced the vanilla autoencoder (AE) employed in [13] and (AnoGan) employed in [2], which was trained on unlabeled data only. During the inference time, the per-pixel error score between the original and reconstructed images was calculated. The defect regions were extracted by thresholding the error map. We selected the threshold value from the value giving the maximum gap between the true positive and false positive from the ROC curve.

For semi-supervised methods, we first evaluated a vanilla Unet [17], which was trained on labeled data only. In addition, we evaluated two generic semi-supervised learning methods, the Π model [23] and MeanTeacher model [24]. Both exploited the unlabeled data and enforced a consistency constraint on the prediction between two different augmentations of input images. We sampled two random augmentations in the same way introduced in Section 3.3 for consistency-based semi-supervised learning. Finally, we evaluated our AE + Unet model by pre-training the AE on all available data and then trained the Unet with limited labeled data.

### 4.4. Evaluation Metric

We used the mean IoU (intersection over union) to evaluate the performance of each model. The IoU for each class is given by

$$IoU=\frac{TP}{TP+FP+FN}$$

where TP, FP, and FN are the numbers of true positive, false positive, and false negative pixels, respectively.

Table 2 reports the results from four experimental settings on fifteen object categories. The Unet trained with few labeled data in a supervised fashion outperformed unsupervised learning approaches by a large margin although it was highly over-fit. The reason is that segmentation labeling provided very strong supervision to the model. Moreover, unsupervised learning methods usually find the difference between the reconstructed result and the input defect sample. The fault localization is aided by the reconstruction error from the texture of the object. The error can be high in an imperfect reconstruction area. Another possible reason is that the selected threshold is not maximized in terms of IoU. A very small false positive rate can have a huge effect on the IoU score. Although the Π model leverages both unlabeled and labeled data, it is worth noting that it only improves performance insignificantly compared to the over-fit Unet-based line, around 0.4% overall. However, it tends to have better results in texture object categories. The model may suffer from highly imbalanced defect pixels versus non-defect pixels in an image.

Overfitting is caused by training a network with a much higher number of parameters compared to the dataset size. Although the network can estimate a complex function, it also fits well to noise with the dataset instead of finding its trend. Since Unet has a lot of parameters, the overfitting effect cannot be avoided with the limitation of the training data size. However, our method adds another image as the network input. This can increase useful data implicitly, thus it can help to mitigate the overfitting effect. The result in Table 3 shows that there was a sizable gap of IoU between evaluation in training and testing datasets. However, the gap was reduced when applying our method as shown by the mean IoU.

**Figure 6:**
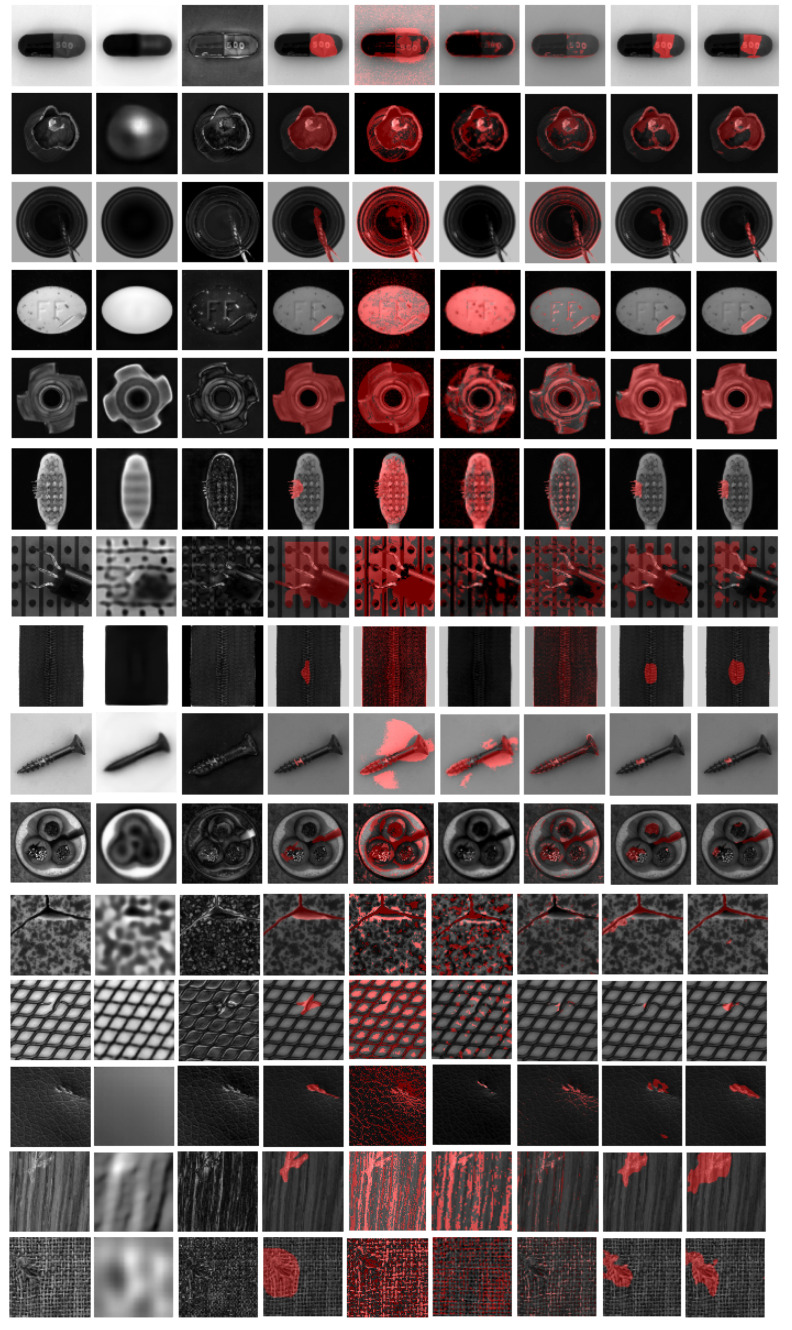
Qualitative results of all object categories. From left to right: original image, reconstructed image from AE, difference image, ground truth, results from PCA, unsupervised AnoGan, unsupervised AE, results from mean teacher, and results from our proposed method.

Our proposed method outperforms substantially compared to the unsupervised autoencoder and fully-supervised Unet baseline. It also beats the Unet Π model, which is trained in semi-supervised learning fashion. This is because our method extracts the useful knowledge to the anomaly detection task. The high values in the difference image indicate potential anomaly pixels. The subsequent Unet learns to take advantages from features in both raw data and difference maps. The experiment shows that the difference maps can enhance the resulting segmentation maps. The qualitative results are shown in Figure 6.

More comprehensive evaluation results are given in Appendix A.

### 4.5. Ablation Study

In this section, we show the results of the ablation study for two scenarios, i.e., ablation study for data augmentation as well as that for feeding different inputs to the network.

From Table 4, it can be noted that data augmentation can bring the result up significantly. Data augmentation increases the amount of data by adding slightly modified copies of already existing data or newly created synthetic data from existing data. With this technique, it helps the model to have more data exposure. The result in Table 4 also shows that different images can assist the network to identify the defect regions. Thus, feeding both original and different images also yields a visibly marked-up IoU score.

## 5. Conclusions

In this work, we proposed a network for semantic anomaly detection. The solution has been designed for costly ground truth obtaining. Due to highly imbalanced defect pixels compared to normal pixels in an image, the conventional semi-supervised methods cannot work well in this setting. We first introduced an autoencoder to extract useful information from unlabeled data by capturing the potential defect pixels as a difference image. Then, the successive segmentation module was trained by this difference image from labeled data along with its original input. The result from the autoencoder helps the segmentation network to suffer less from over-fitting and instability due to a very small training set. The experimental results show a reduced gap between evaluation of the training and testing datasets. Moreover, they provide guidance about possible defect pixels.

The experimental results show that the proposed strategy improves the performance substantially in certain object categories and the overall result from all categories is increased by approximately a 3% margin. In this work, we employed Unet as a choice for the segmentation network. However, the choice of network architecture can be changed accordingly in different conditions. The limitation lies in the challenge of the defect appearance. The shape of some faults is quite thin and small. The model will have difficulty in detecting them as a result of this.

## Figures and Tables

**Table 1:** Number of unlabeled, labeled, and test samples in the dataset for each category of the MVTec-AD dataset.

**Category**	Bottle	Cable	Capsule	Hazelnut	Metal nut
**Unlabeled**	217	242	231	401	230
**Labeled**	4	9	6	5	5
**Test**	71	123	114	95	100
**% Labeled**	1.80	3.58	2.53	1.23	2.12
**Category**	Pill	Screw	Toothbrush	Transistor	Zipper
**Unlabeled**	283	332	64	223	256
**Labeled**	8	6	2	5	8
**Test**	143	142	36	85	127
**% Labeled**	2.74	1.77	3.03	2.19	3.03
**Category**	Carpet	Grid	Leather	Tile	Wood
**Unlabeled**	292	276	257	242	259
**Labeled**	6	6	6	6	6
**Test**	396	240	424	396	244
**% Labeled**	2.02	2.12	2.28	2.42	2.26

**Table 2:** Semantic segmentation performance of several evaluated methods. We report the IoUs of unsupervised PCA, unsupervised AnoGan, unsupervised autoencoder (AE), Unet, pi models (Π*), mean teacher, and our method (AE + Unet).

Category	PCA	AnoGan	AE	Unet	Π*	Mean Teacher	AE + Unet
Bottle	0.131	0.008	0.543	0.606	0.605	0.740	0.655
Cable	0.090	0.001	0.376	0.696	0.695	0.665	0.695
Capsule	0.048	0.023	0.480	0.545	0.544	0.557	0.579
Hazelnut	0.087	0.067	0.437	0.791	0.791	0.769	0.791
Screw	0.006	0.008	0.470	0.587	0.585	0.664	0.589
Pill	0.090	0.075	0.520	0.631	0.650	0.579	0.759
Metal Nut	0.194	0.172	0.519	0.866	0.866	0.568	0.888
Toothbrush	0.070	0.038	0.486	0.544	0.544	0.574	0.542
Transistor	0.102	0.071	0.436	0.560	0.530	0.563	0.605
Zipper	0.035	0.001	0.455	0.722	0.720	0.698	0.730
Grid	0.009	0.008	0.469	0.533	0.561	0.500	0.636
Carpet	0.017	0.020	0.381	0.713	0.630	0.700	0.681
Leather	0.017	0.016	0.462	0.673	0.676	0.666	0.635
Tile	0.096	0.065	0.477	0.603	0.746	0.714	0.674
Wood	0.070	0.057	0.502	0.591	0.570	0.761	0.652
**Mean Total**	0.071	0.042	0.467	0.644	0.647	0.6478	0.666

**Table 3:** Evaluation of the overfitting effect between Unet and our method. We report the IoUs of the results from both training and testing data.

Category	Unet		AE + Unet	
	Train Data	Test Data	Train Data	Test Data
Bottle	0.980	0.597	0.987	0.655
Cable	0.968	0.628	0.968	0.695
Capsule	0.938	0.553	0.940	0.579
Hazelnut	0.981	0.785	0.980	0.791
Screw	0.940	0.569	0.945	0.589
Pill	0.926	0.597	0.931	0.759
Metal Nut	0.990	0.857	0.990	0.888
Toothbrush	0.968	0.481	0.984	0.542
Transistor	0.984	0.558	0.983	0.605
Zipper	0.966	0.721	0.967	0.730
Grid	0.915	0.580	0.921	0.636
Carpet	0.969	0.598	0.969	0.681
Leather	0.956	0.615	0.955	0.635
Tile	0.966	0.634	0.966	0.674
Wood	0.978	0.579	0.978	0.652
**Mean Total**	0.961	0.623	0.964	0.666

**Table 4:** Ablation study of data augmentation and different types of input. We report the IoUs of feeding the original image with and without data augmentation, the augmented different image, and the augmented original image combined with the different image.

Category	w/o Data Aug	Data Aug	Diff	Original +Diff
Bottle	0.583	0.597	0.637	0.655
Cable	0.620	0.628	0.642	0.695
Capsule	0.565	0.553	0.555	0.579
Hazelnut	0.766	0.785	0.701	0.791
Screw	0.590	0.569	0.559	0.589
Pill	0.607	0.597	0.579	0.759
Metal Nut	0.805	0.857	0.882	0.888
Toothbrush	0.478	0.481	0.532	0.542
Transistor	0.574	0.558	0.587	0.605
Zipper	0.731	0.721	0.672	0.730
Grid	0.563	0.580	0.584	0.636
Carpet	0.381	0.598	0.619	0.681
Leather	0.607	0.615	0.666	0.635
Tile	0.595	0.634	0.669	0.674
Wood	0.557	0.579	0.608	0.652
**Mean Total**	0.614	0.623	0.632	0.666

## Data Availability

The datasets generated during and/or analyzed during the current study are available from the MVTec website, https://www.mvtec.com/company/research/datasets/mvtec-ad (accessed on 3 May 2020).

## Appendix A

### Appendix A.1. We Provide the Average Process Time in for Each Object Class

**Table A1:** Semantic segmentation performance of several evaluated methods. We report average processing time in milliseconds of unsupervised PCA, unsupervised AnoGan, unsupervised autoencoder (AE), Unet, pi models (Π*), mean teacher, and our method (AE + Unet). Only PCA was executed on the CPU. Other methods were run on a GPU. It turns out that Unet, pi mean teacher, and AE + Unet have no significant difference in processing time.

Category	PCA	AnoGan	AE	Unet	Π*	Mean Teacher	AE + Unet
Bottle	67	19	25	55	52	49	51
Cable	106	15	16	45	42	44	38
Capsule	99	16	17	49	44	39	40
Hazelnut	90	16	19	53	49	45	43
Screw	106	19	15	44	41	42	36
Pill	116	15	14	45	43	39	36
Metal Nut	89	17	20	50	47	42	41
Toothbrush	53	21	44	54	56	58	53
Transistor	85	16	20	56	54	49	46
Zipper	107	15	15	34	41	44	38
Grid	66	19	10	37	35	35	29
Carpet	106	16	17	32	29	31	26
Leather	107	16	17	32	30	32	26
Tile	91	15	17	32	30	27	26
Wood	78	17	11	36	34	30	29
**Mean Total**	91	17	18	43	40	40	37

### Appendix A.2. Mean Absolute Error for Each Object Class. We Compute the Mean of the Sum of All the Pixels That Are Incorrectly Classified

**Table A2:** Semantic segmentation performance of several evaluated methods. We report the mean absolute error (MAE) of the unsupervised PCA, unsupervised AnoGan, unsupervised autoencoder(AE), Unet, pi models (Π*), mean teacher, and our method (AE + Unet).

Category	PCA	AnoGan	AE	Unet	Π*	Mean Teacher	AE + Unet
Bottle	59,216	968	25,138	2952	2833	2096	2485
Cable	38,224	496	10,784	21,925	1154	2039	1392
Capsule	22,466	1717	17,787	1002	587	927	634
Hazelnut	32,664	3171	2321	1548	1406	665	678
Screw	48,335	2964	1672	345	224	177	172
Pill	49,431	5695	5379	2871	1892	1671	1481
Metal Nut	82,348	6200	4809	12,105	7593	6653	1595
Toothbrush	32,227	4887	1042	5273	1875	1258	925
Transistor	49,926	4403	2979	15,916	2655	2018	1868
Zipper	71,271	346	3577	7624	859	868	1278
Grid	63,086	1751	1240	2745	439	450	379
Carpet	78,140	4275	1578	2321	664	665	1023
Leather	46,093	1096	879	558	311	342	319
Tile	26,854	4123	4457	3074	4617	2779	2522
Wood	44,790	5188	4698	3524	7826	1339	8731
**Mean Total**	47,494	3152	5889	5585	2329	1596	1698
